# Optimizing Camera Exposure Time for Automotive Applications

**DOI:** 10.3390/s24165135

**Published:** 2024-08-08

**Authors:** Hao Lin, Darragh Mullins, Dara Molloy, Enda Ward, Fiachra Collins, Patrick Denny, Martin Glavin, Brian Deegan, Edward Jones

**Affiliations:** 1 School of Engineering, University of Galway, University Road, H91 TK33 Galway, Ireland; 2 Ryan Institute, University of Galway, University Road, H91 TK33 Galway, Ireland; 3 Valeo Vision Systems, Tuam, Co., H54 Y276 Galway, Ireland; 4 Computer Science and Information Systems (CSIS), Faculty of Science and Engineering, University of Limerick, Castletroy, V94 T9PX Limerick, Ireland

**Keywords:** image quality, computer vision, object detection, low light conditions, ADAS, autonomous vehicles

## Abstract

Camera-based object detection is integral to advanced driver assistance systems (ADAS) and autonomous vehicle research, and RGB cameras remain indispensable for their spatial resolution and color information. This study investigates exposure time optimization for such cameras, considering image quality in dynamic ADAS scenarios. Exposure time, the period during which the camera sensor is exposed to light, directly influences the amount of information captured. In dynamic scenarios, such as those encountered in typical driving scenarios, optimizing exposure time becomes challenging due to the inherent trade-off between Signal-to-Noise Ratio (SNR) and motion blur, i.e., extending exposure time to maximize information capture increases SNR, but also increases the risk of motion blur and overexposure, particularly in low-light conditions where objects may not be fully illuminated. The study introduces a comprehensive methodology for exposure time optimization under various lighting conditions, examining its impact on image quality and computer vision performance. Traditional image quality metrics show a poor correlation with computer vision performance, highlighting the need for newer metrics that demonstrate improved correlation. The research presented in this paper offers guidance into the enhancement of single-exposure camera-based systems for automotive applications. By addressing the balance between exposure time, image quality, and computer vision performance, the findings provide a road map for optimizing camera settings for ADAS and autonomous driving technologies, contributing to safety and performance advancements in the automotive landscape.

## 1. Introduction

Digital camera systems have been used by humans for decades, and such systems have primarily been tuned and calibrated for human vision. With the increasing use of cameras in computer vision and autonomous vehicle systems, it is now important to consider the different effects that digital camera configuration has on automotive applications where computer vision may be an important component of such a system; in particular, a good quality image for human vision may not be equally as good for computer vision. Studies have already shown that image enhancement algorithms that make images more appealing for human vision do not improve computer vision performance [1,2].

There are many factors that limit performance in autonomous vehicle computer vision systems, including the environment, hardware, and software. The environment includes the target, the target background, and the path between the image sensor and the target [3]. Hardware factors include the characteristics of the camera lens and the image sensor itself [4]. Software factors include any data post-processing performed on the image generated by the sensor, including the Image Signal Processor (ISP) and image processing algorithms [5].

RGB cameras are widely used in automotive applications, providing low-cost and scalable solutions. One of the challenges of automotive camera systems that RGB cameras have difficulty with is that they have to operate across multiple lighting conditions. This demands clear and accurate image capture under varying light conditions, such as transitioning from bright daylight to the shadows of a tunnel. Another challenge is mitigating the effects of motion blur caused by the relative motion between the vehicle and surrounding objects. Additionally, the automotive industry demands robust image quality metrics that can predict and improve the performance of computer vision algorithms. Traditional metrics like Modulation Transfer Function (MTF) and Signal to Noise Ratio (SNR), while helpful, do not fully capture the complexities of real-world driving conditions and their impact on computer vision performance, though such standard metrics are commonly used in the camera manufacturing process [6]. Therefore, developing and validating new metrics tailored to automotive applications is essential.

In a camera processing pipeline, there is typically an ISP pipeline, which converts a raw image signal from an image sensor into a viewable format [7]. The ISP pipeline includes steps such as denoising and demosaicing [5,8]. ISPs in cameras are generally tuned for human perception [5]. An important parameter in a camera system is the exposure time of the camera, i.e., the amount of time the image sensor is capturing the image. The longer the exposure time, the more photons are captured. Having an adequate exposure time is crucial for camera systems, as an underexposed image will have high noise and poor contrast, whereas an overexposed image will saturate, resulting in information loss for the brightest parts of an image’s scene. The question of what the optimal exposure time is depends on the application and the capture environment, i.e., the optimal exposure time for a daytime (bright) environment will not be the same as the optimal exposure time for a night-time (dark) environment. Similarly, the optimal exposure time for human vision and computer vision may also be different.

Another issue with extending the exposure time is motion blur. Motion blur is caused by the relative motion between the camera and the target during the capture window. The degree of motion blur is a function of the exposure time as well as the relative velocity of the camera and the objects in the scene [9].

Traditionally, the quality of the image for computer vision systems has been measured by Key Performance Indicators (KPIs) such as MTF and SNR. MTF measures the sharpness in the scene and SNR compares the signal to the background noise (see Section 2.2). These traditional metrics alone, although useful, are not sufficient to describe image quality for computer vision. Previous research has shown that the relationship between metrics such as MTF and object detection performance is not strong [10]. Despite this, MTF is still used by the industry as a key image quality metric in many camera-based systems. In this paper, the authors explore the correlation between exposure time, motion blur, and image quality, and investigate the effect of motion blur and exposure time on object detection performance, specifically in the context of automotive applications. The contributions of the paper are as follows:

1. A physics-based simulation tool to generate samples test images that models pixel noise, ambient lighting, exposure time, optical blur, and motion blur, which extends previous work by the authors. The simulation software is available at https://github.com/HaoLin97/Motion_Blur_Simulation.
2. An analysis of the effects of motion blur and exposure time on a range of Image Quality (IQ) metrics, including widely-used metrics such as MTF and SNR, as well as additional metrics.
3. Analysis of the efficacy of image quality metrics as predictors of motion blur and also their correlation with object detection performance.
4. A detailed investigation of Optical Character Recognition (OCR) and object detection on stop signs with different exposure times and different degrees of motion blur.
5. A set of methodologies to optimize exposure time for a given scene illumination and relative motion.

This paper is organized as follows: first, background and related works are discussed in Section 2. Section 3 covers the simulation software for generating test images, which is based on previous work by the authors, as well as the associated test methodology for analyzing the performance of image quality metrics when the image is affected by various degrees of motion blur and exposure time. The correlation between exposure time and motion blur with image quality metrics and OCR performance is discussed in Section 4. Section 6 contains the conclusions.

## 2. Background and Related Works

### 2.1. Camera Configurations

The evolution of automotive cameras has taken two distinct routes, one of which provides an image for human vision purposes and the other provides an image to an Advanced Driving Assistance System (ADAS) for machine vision purposes. These two have inherently different requirements.

Image quality for human vision is largely subjective, and this area has been greatly explored by researchers and practitioners. Early automotive cameras were developed for human vision for applications such as reverse cameras, side-view, and surround-view fisheye cameras [11]. Cameras in conjunction with other sensors allow the driver to be more aware of the surroundings.

Awareness of the environment is also necessary for autonomous vehicles to perform safety-critical processes when driving on the roads, such as object detection, trajectory prediction, and object avoidance [12]. In parallel with the development of computer vision algorithms, automotive camera systems have been influenced by such developments, e.g., through processes such as geometric calibration and compensating for lens distortion. These calibration steps are generally conducted before ISP tuning.

#### 2.1.1. Image Signal Processor (ISP)

Converting a raw signal from an image sensor to a viewable format includes various steps such as demosaicing, denoising, and gamma control, collectively referred to as ISP. Different ISP combinations have different effects on the resulting image. It is as yet uncertain if ISP tuning that benefits human vision will also benefit computer vision. Yahiaoui et al. [13] proposed that there is potential to optimize the settings of an ISP that could improve the performance of computer vision algorithms. Molloy et al. [5] characterized the performance degradation of Convolutional Neural Network (CNN) based object detection models due to varying ISP parameters and noted that not all CNN architectures experience the same degree of performance degradation due to ISP parameters.

#### 2.1.2. Noise Sources

In digital imaging, there are multiple sources of noise. These include photon response non-uniformity (PRNU), photon shot noise, offset fixed-pattern noise, dark-current shot noise, demosaicing, digital filtering, and quantization noise [14].

Some of these noise sources are independent of the illumination of the environment. Dark noise or thermal noise occurs as a result of the thermal energy in the environment triggering the camera’s CMOS sensor, leading to current generated at pixels where photons may not have been captured. This noise is proportional to the exposure time and in cameras typical of the state of the art, doubles with every 8 °C increase in temperature [14,15]. The offset fixed-pattern noise (FPN) is the innate noise that is generated by the sensor itself, and this is caused by fabrication error.

Some noise sources are affected by the environment’s illumination; these include photon shot noise and photon response non-uniformity. Photon shot noise is the error caused during the capture of photons due to the number of photons incident on the image sensor varying for any given time interval; this variation follows a Poisson distribution. The signal-to-noise ratio for photon shot noise is the square root of the number of photons captured.

Other noise sources are caused by the digital processing component of the camera, including quantization noise, caused during the conversion from analog voltage reading to digital values.

#### 2.1.3. Exposure and Motion Blur

Exposure time is one of the most important parameters when configuring the camera. It directly correlates to the amount of photons being captured by the sensor. With longer exposure times, more photons are captured, leading to higher SNR. However, longer exposure time does not necessarily guarantee better images.

The signal level in an image will generally increase with exposure time; similarly, noise will also increase with exposure time. As shown in Equation (1) below, the SNR is proportional to the squared root of the exposure time (*T*). For dynamic scenes, motion blur is a function of exposure time, so it can be noted that motion blur is directly proportional to time, shown in Equation (2).

(1) $$SNR\propto \sqrt{T}$$

(2) MotionBlur∝T

With longer exposure time, there is a higher risk of the image becoming overexposed, whereby the sensor is saturated with photons and the pixels are no longer providing useful information. The contrast between the overexposed pixel and the neighboring pixels begins to diminish as overexposure occurs.

As the exposure time increases, the time allowed for motion to take place also increases. Also, the faster the camera or the objects in the scene move, the greater the degree of motion blur. Furthermore, the closer the target is to the camera, the larger the object in the image; consequently, when the target moves, more motion-blurred pixels are observed.

Many studies have shown that blur is one of the most destructive forms of image deformation [16,17,18,19]. Studies have also shown that object detection performance is significantly affected by blur. However, many of these studies use Gaussian blur kernels and do not fully investigate the effects of motion blur, which will be more common in automotive applications [9]. A review of leading motion blur studies indicates that exposure time is not generally taken into consideration [16,17,18,19]. This paper addresses this gap.

In addition to motion blur, other forms of blur include Gaussian blur [20], lens blur, and defocus blur. These types of blur also prove to have a negative impact on object detection performance [5,19]. While deblurring algorithms can be effective on some types of blur, motion blur is often neglected in computer vision research. Zheng et al. [21] proposed a one-stage deblurring framework that integrates deblurring with object detection. Nayar et al. [22] have modeled blur via the Point Spread Function(PSF) to obtain the parameters for deblurring. Hu et al. [23] developed a method that uses light streaks in a blurred image to assist the deblurring kernel. Although these deblurring algorithms may improve object detection performance, they are often computationally demanding which limits their real-time utility.

At the same time, Argaw et al. [24] have proposed that there may be value in motion blur existing in images, for example, in optical flow estimation and video sequence restoration.

### 2.2. Image Quality Metrics

Image quality metrics in common usage in camera systems include MTF [25,26] and SNR [27]. As noted earlier, such metrics have limitations when used in challenging imaging applications, particularly in terms of their correlation with object detection performance. Recently-proposed metrics such as Shannon Information Capacity (SIC) [28] and Noise Equivalent Quanta (NEQ) [29], which combine signal and noise components in different ways, are increasing in popularity.

There are also other types of metrics including full-reference image quality metrics such as Mean Squared Error (MSE), Peak Signal-to-Noise Ratio (PSNR), Structural Similarity Indexing Method (SSIM) and Feature Similarity Indexing Method (FSIM) [30,31,32,33]. These full-reference image quality metrics are not suitable for automotive applications as they generally require a high-quality reference image, which is often not possible in practical applications, particularly automotive applications with highly dynamic scenes because there is constant change in noise and blur in the image, both of which will impact the image quality.

A list of the metrics considered in this study, and their units, is given in Table 1.

#### 2.2.1. MTF and SNR

The difference between bright and dark elements in an image is referred to as contrast [26]. The MTF captures the contrast preserved by an imaging system, as a function of spatial frequency, and is an important measure of sharpness [25]. Michelson contrast [34] (C(f)) and MTF, at a spatial frequency *f* and luminance *V*, are calculated as shown in Equations (3) and (4) [35].

(3) $$C\left(f\right)=\frac{V_{max}-V_{min}}{V_{max}+V_{min}}$$

(4) $$MTF\left(f\right)=100\%*\frac{C(f)}{C(0)}$$

where $V_{max}$ and $V_{min}$ are the maximum and minimum luminance levels in the image. MTF50 is a commonly used summary metric for cameras and is defined as the point on the MTF curve where the contrast decays to 50% of its low-frequency values. MTF50 is also being considered as a standard metric by the IEEE P2020 Committee that is examining image quality for automotive applications [36]. SNR is a well-known measure that compares the level of a desired signal to the level of background noise [27]. There are 2 types of SNRs used in this study, Patch SNR and Edge SNR. Note, that in this study Patch SNR is expressed as a ratio and Edge SNR is expressed as Decibels(db). Patch SNR is calculated using:

(5) $$SNR=\frac{\mu }{\sigma }$$

where μ is the mean and σ is the standard deviation of the pixel values in a square patch, of size 240 px × 240 px, in the center of the image.

The Edge SNR [37] is calculated from the information of a slanted edge test image, using Equation (6):

(6) $$SNR_{Edge}=20log_{10}\left(\frac{S}{N}\right)$$

where *S* is the signal component and *N* is the Noise component.

**Figure 1:**
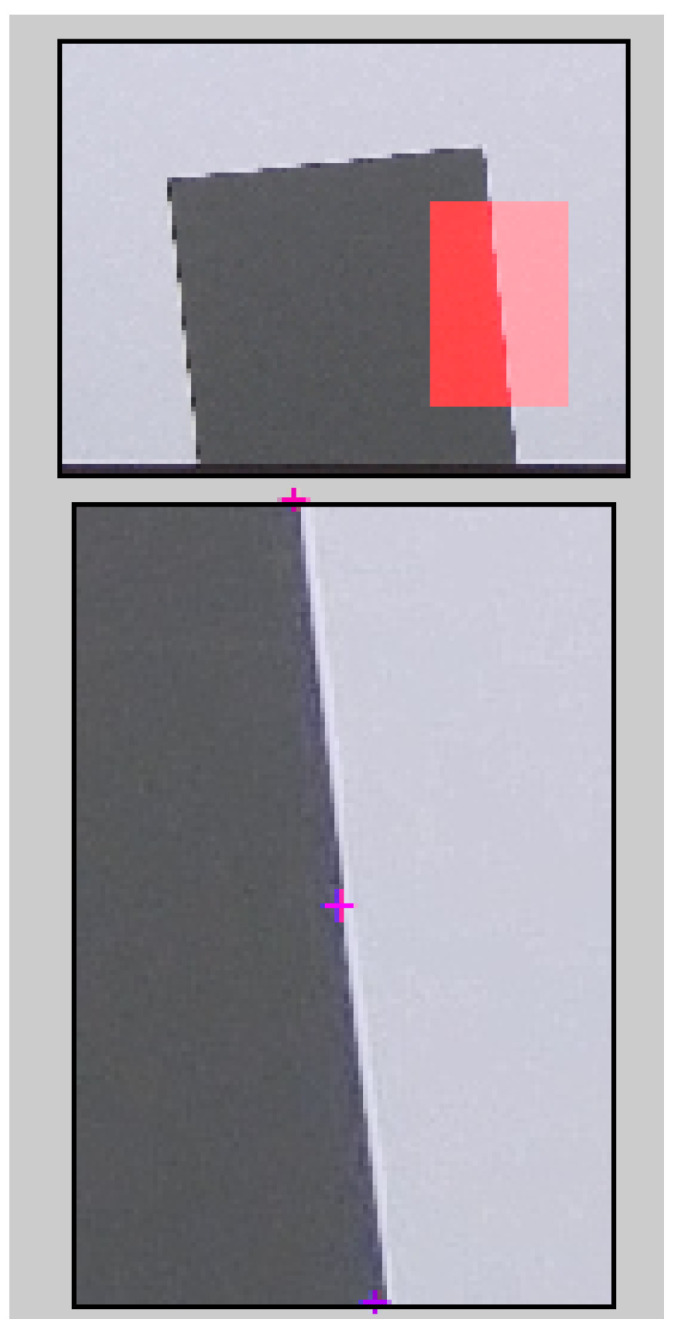
Example of an edge (highlighted in red) used for calculation on the slanted edge chart [38]. The bottom image is an enlarged version of the red highlighted area in the top image.

An example of a slanted edge test image, from which Edge SNR information is extracted, is shown in Figure 1. The signal and noise information are extracted from the edge according to the method described in [38].

#### 2.2.2. Shannon Information Capacity (SIC)

SIC [28,39] is widely used in communication systems and information theory. It defines the maximum rate in bits per second at which data can be transmitted without error through a communications channel. From an image quality perspective, a camera may be viewed as a “channel”. As a result, SIC can be viewed as a measure of the maximum “information” that an image contains. As shown in Equation (7) [28], SIC combines MTF and SNR, where S(f) is the signal power spectrum. N(f) is the noise power spectrum, and *B* is the Nyquist frequency:

(7) $$SIC=2\pi \int_{0}^{B}log_{2}\left(\frac{S(f)+N(f)}{N(f)}\right)fdf$$

#### 2.2.3. Noise Equivalent Quanta (NEQ)

The calculation of NEQ, as a function of spatial frequency, combines MTF and the Noise Power Spectrum (*N*) [28,29]. In Equation (8), μ is the mean of the signal and *v* is the spatial frequency.

(8) $$NEQ\left(v_{x},v_{y}\right)=\frac{MTF^{2}\left(v_{x},v_{y}\right)}{N(v_{x},v_{y})/\mu }$$

### 2.3. Object Detection

Image object detection algorithms have advanced significantly in recent years, and have become more resistant to many sources of degradation, including rotation, noise, and blur [40,41]. Convolutional Neural Networks (CNN) are well established in objection detection tasks; however, in recent years, vision transformers have challenged CNNs as the state of the art for such applications [42,43,44].

Classical CNN-based detectors can be divided into two categories: two-stage detectors and one-stage detectors. In two-stage detectors, the first stage is used to generate a region of interest, and the second stage classifies that region of interest according to the object classes. One-stage detectors propose prediction boxes directly without the region proposal step and hence are faster and more suited for real-time applications, but they sacrifice some accuracy for speed. Two-stage approaches, such as the Region-based Convolutional Neural Network (R-CNN) [45], Faster-R-CNN [46] and mask-R-CNN [47] have better localization and better accuracy. One-stage approaches such as Single Shot MultiBox Detector (SSD) [48] and You Only Look Once (YOLO) [49,50,51,52] achieve faster inference speeds.

In general, object detection performance degrades under adverse environmental conditions [3], including low light, rain, fog, and snow, to different degrees. For example, in low-light conditions, there are complications such as glare, noise, and imbalanced lighting. Kennerley et al. [53] proposed a domain adaptive framework that attempts to bridge the differences between day and night data through data augmentation.

Liu et al. [54] attempted to quantify the performance of automotive perception systems. In their work, they showed that mean average precision (mAP) increases with MTF50 and decreases with object distance. The MTF50 was varied through different combinations of camera aperture size settings and sensor pixel sizes.

Classic object detection algorithms are very sensitive to various types of blur [16,17,18,19]. When an image is blurred, the object loses its sharpness, and consequently, object edges become more difficult to identify. Salas et al. [55] suggested that fine-tuning a pre-trained model with blurred images can improve its object detection performance.

## 3. Simulation

### 3.1. Overview

**Figure 2:**
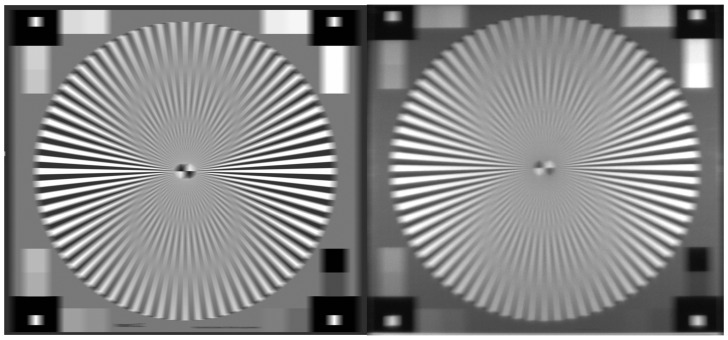
Simulated blurred image on the left and real blurred image on the right of a Siemens Star chart at 30 ms exposure time, 200 lux illumination, and 1500 Horizontal Pixel Movement Per Second. Similar artifacts are seen in both images.

As part of this study, a simulation tool was developed to simulate RGB images with different exposure times, ambient lighting, and motion blur. The metric for motion blur used in this study and in the software is pixel movement per second. This study mainly focuses on *horizontal pixel movement per second* (DX). The simulation tool allows for quick and efficient exploration of the test space. An example of a real motion-blurred image versus a simulated motion blur image is shown in Figure 2. The simulation has some assumptions (1) it assumes a monochromatic scene (2) it assumes a global shutter capture mode (3) it uses a simplified lens model. This software includes a comprehensive noise model, which extends the works of [56,57,58,59].

This simulation tool is an improved version of the authors’ previous works [60]. In this updated version, the noise model and the lighting model are updated to follow the EMVA1288 standard [61], allowing for more realistic physics-based simulation images.

In conjunction with the main simulation software, a trigonometric tool (described in Section 3.5) was also developed for the calculation of the expected motion blur from the target speed and distance between the target and the camera.

**Figure 3:**
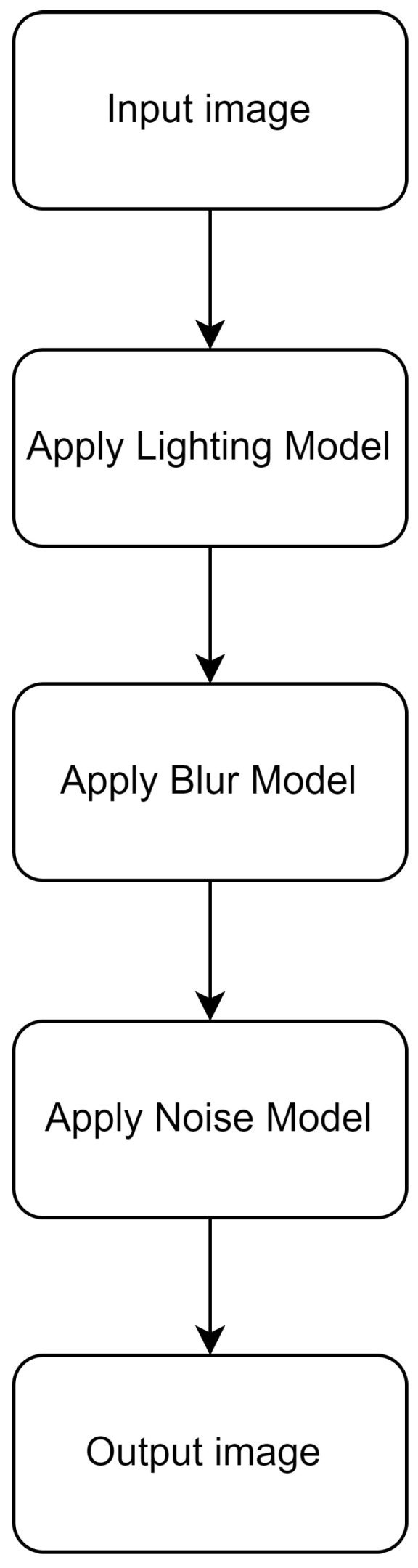
Simulation flowchart.

As shown in Figure 3, the input to the simulation is an image and a set of parameters that include camera configurations, ambient lux level, degree of motion, and exposure time. The image is converted to grayscale, following which a background mask at an 18% gray level is applied, to facilitate the blur operation. This is followed by optical blurring, and motion blur, followed by the noise model. Finally, the image is then converted to digital units based on the camera configuration values.

The camera configuration values that are used include the quantum efficiency, sensitivity, baseline offset, and bit depth of the camera. The details of the simulation model are explained further below.

### 3.2. Light Model

The number of photons hitting the sensor is calculated using the ambient lux level. Lux is used as an input metric because it is a widely recognized quantity; however, its photometric nature makes it unsuitable for calculations. Lux is converted to its radiometric counterpart (W/m^2^) and then converted to photons to be used within the simulation software. The calculations used for the conversion are an extension of those described in Jenkin et al. [58].

(9) $$E_{SOURCE}=683.\int_{\lambda_{MIN}}^{\lambda_{MAX}}W\left(\lambda \right)V\left(\lambda \right)d\lambda$$

The source light level $E_{SOURCE}$ is defined in Equation (9), where $\lambda_{MAX}$ and $\lambda_{MIN}$ are the maximum and minimum wavelengths of interest. V(λ) is the relative spectral luminous efficiency curve according to the Commission Internationale de l’Éclairage (CIE) standard [62] scaled by the peak luminous efficacy of human vision (683 lumens per watt at 555 nm). As shown in Equation (10), the scaled light level ($E_{SCALE}$) is the ambient light level ($E_{AMB}$) divided by the source light level.

(10) $$E_{SCALE}=\frac{E_{AMB}}{E_{SOURCE}}$$

The energy per photon, ϵ(λ), can be calculated using Equation (11), where *h* is Planck’s constant, 6.62 × 10^−34^ m^2^ kg s^−1^, and *c* is the speed of light, 299,792,458 ms^−1^.

(11) $$\epsilon \left(\lambda \right)=\frac{hc}{\lambda }$$

The power per nm per pixel, $P_{p}\left(\lambda \right)$, can be calculated using Equation (12):

(12) $$P_{p}\left(\lambda \right)=\frac{E_{SCALE}}{\pi }W\left(\lambda \right)I\left(\lambda \right)Q\left(\lambda \right)\Omega_{L}t_{o}A_{p}$$

where W(λ) is the CIE D55 illumination curve, $\Omega_{L}$ is the solid angle of the lens, I(λ) is the infrared cut off filter, Q(λ) is the quantum efficiency curve, $\Omega_{L}$ is the solid angle of the lens, $t_{o}$ is the transmission loss, $A_{p}$ is the area of the pixel. As shown in Equation (13), dividing $P_{p}\left(\lambda \right)$ by ϵ(λ), multiplying by the integration time, $T_{INT}$, and integrating over wavelength yields the total number of photoelectrons captured by the pixel, $PE_{p}$:

(13) $$PE_{p}=\int_{\lambda_{MIN}}^{\lambda_{MAX}}\frac{T_{INT}.P_{p}\left(\lambda \right)}{\epsilon (\lambda )}d\lambda$$

### 3.3. Blur Model

**Figure 4:**
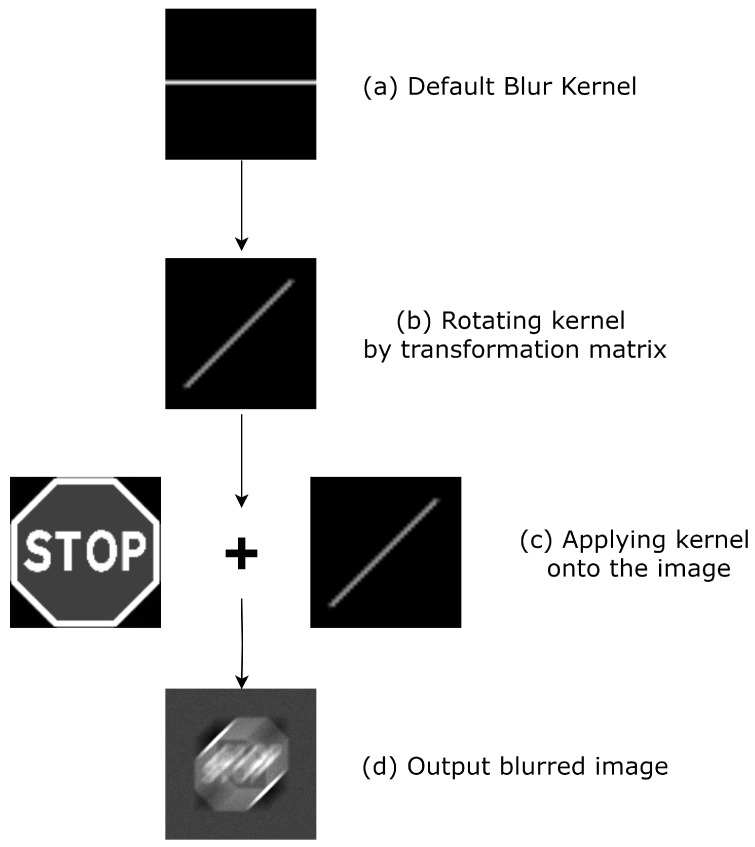
Example of use of blur model.

Optical blurring was modeled using Matlab’s *disk* function. The disk function is chosen because the circular averaging filter used is a better representation of defocus blur than a 2D Gaussian filter [63]. The disk filter simulates the dispersion effects of the lens on the camera. It accounts for the circular shape of the defocus point spread function (PSF) and is more appropriate for blurring that occurs due to changes in the lens focus [63]. Figure 4 shows the steps in the blurring operation. A blur kernel is first created (Figure 4a) based on the vector combination of the horizontal and vertical pixel movements, producing the length of the motion. The kernel is then rotated by a transformation matrix generated by the angle between the horizontal and vertical directions, giving the direction of the blur (Figure 4b). The blur kernel is then normalized and applied to the image (in this example, a STOP sign) as a filter, as illustrated in (Figure 4c,d).

### 3.4. Noise Model

**Figure 5:**
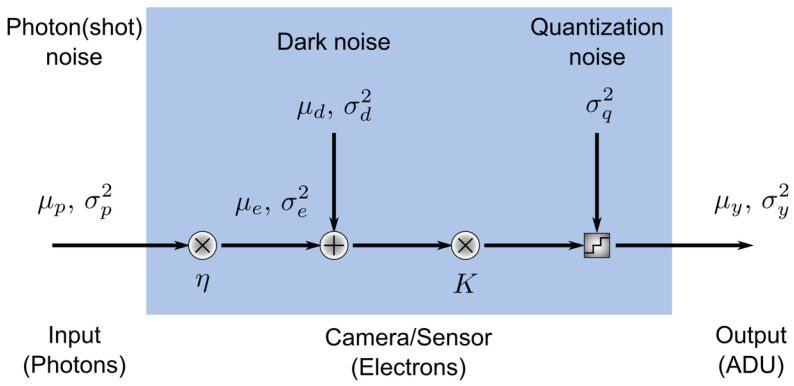
Simulation noise model.

The noise model is shown in Figure 5, where μ and $\sigma^{2}$ represent the mean and variance of, respectively, the number of photons hitting the camera (p), the dark noise (d), and the output signal in analog-digital units (ADU) (y) [56]. The number of electrons $\mu_{e}$ generated by $\mu_{p}$ photons impinging on the active area of the sensor depends on the quantum efficiency η, a property of the camera that in general depends on wavelength.

The noise model used in this study is an extension of the model described in [56], based on the EMVA1288 standard [61]. This converts the input image into photons using the quantum efficiency and sensitivity of the camera. The number of electrons generated by photons impinging on the active area of the sensor depends on the quantum efficiency. The sensitivity of the camera represents the scaling of the resulting voltage.

Shot noise following a Poisson distribution is then added, and the image is then converted to electrons. Dark noise, following a normal distribution, is added to the image.

The image is then converted to ADUs, representing quantization in the camera, to produce the output image. Where applicable to the camera sensor, a baseline offset is applied before outputting the image. The baseline offset is the camera’s built-in baseline ADU value; this prevents the sensor from reading a negative value when the input signal is very low.

The analysis shown in Section 4 is executed on images outputted from the noise model pipeline, i.e., the output(ADU) shown in Figure 5.

### 3.5. Trigonometric Tool

Using a trigonometric tool developed as part of this work (available at the Github repository along with the main simulation software), the theoretical motion blur can be calculated. The tool utilizes trigonometry to calculate the theoretical motion blur, in units of pixels:

(14) $$MotionBlur=\frac{Size_{pixel}\times Speed\times ExposureTime}{Size_{metre}}$$

**Figure 6:**
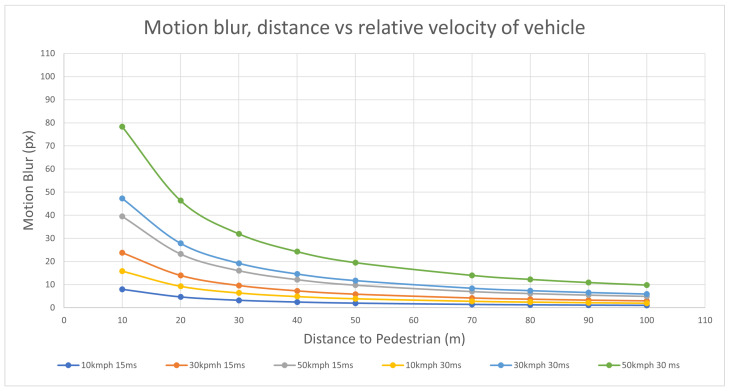
Graph showing the relationship between the number of blur pixels vs. distance at various speeds.

To illustrate: given a 4096 × 2160 camera resolution with a 99 °C horizontal and 63° vertical field of view, yields a 41-pixel per degree representation in the image. Assume a target of size of 4.9 m long and 1.8 m tall, with a speed of 50 kph/13.8 ms^−1^, and a 30 ms exposure time in the camera. The theoretical motion blur is calculated as 9.84 pixels using Equation (14). More examples are shown in Table 2. The relationship between motion blur, distance, and speed is shown in Figure 6.

### 3.6. Methodology

**Figure 7:**
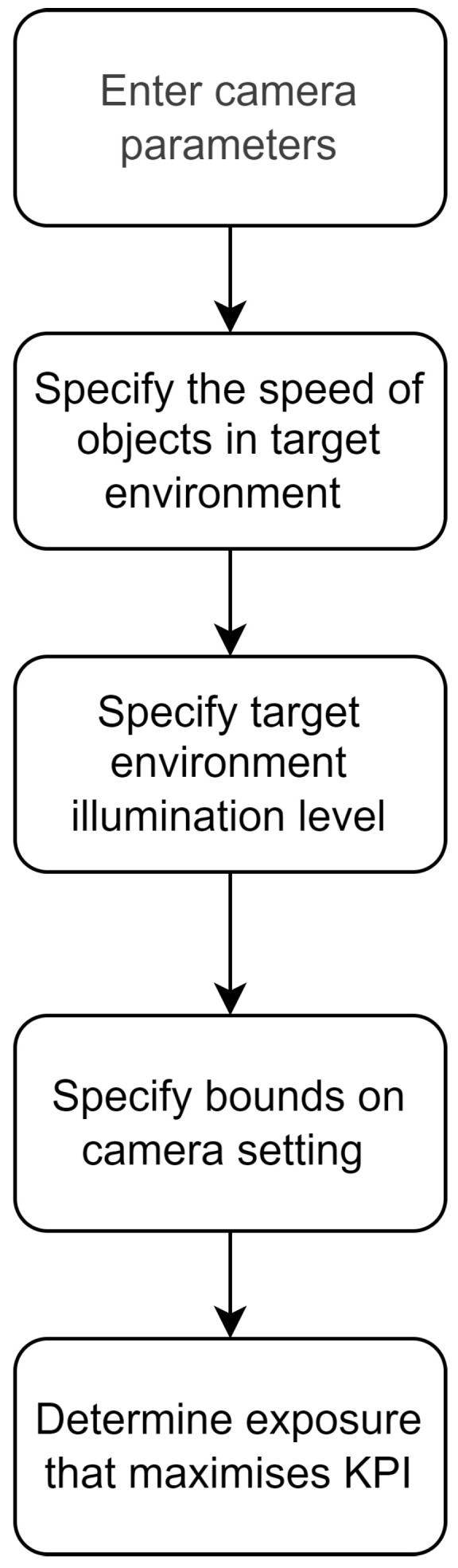
A flowchart for the methodology of finding the optimal exposure time for a given target environment.

Based on the description of the simulation in the preceding sub-sections, Figure 7 shows a flowchart for the overall process of choosing the optimal exposure time. Before beginning the simulation, the input parameters need to be specified. The first step is to specify the camera characteristics for the simulation. This includes the camera’s quantum sensitivity, quantum efficiency, bit depth, saturation capacity, and baseline offset. The next step is to specify the speed of objects in the target environment.

The following step is to configure the environmental component of the simulation, including choosing the illumination spectrum of the simulated light sources, for example, monochromatic lights. The ambient light level will have to be defined, in lux.

**Figure 8:**
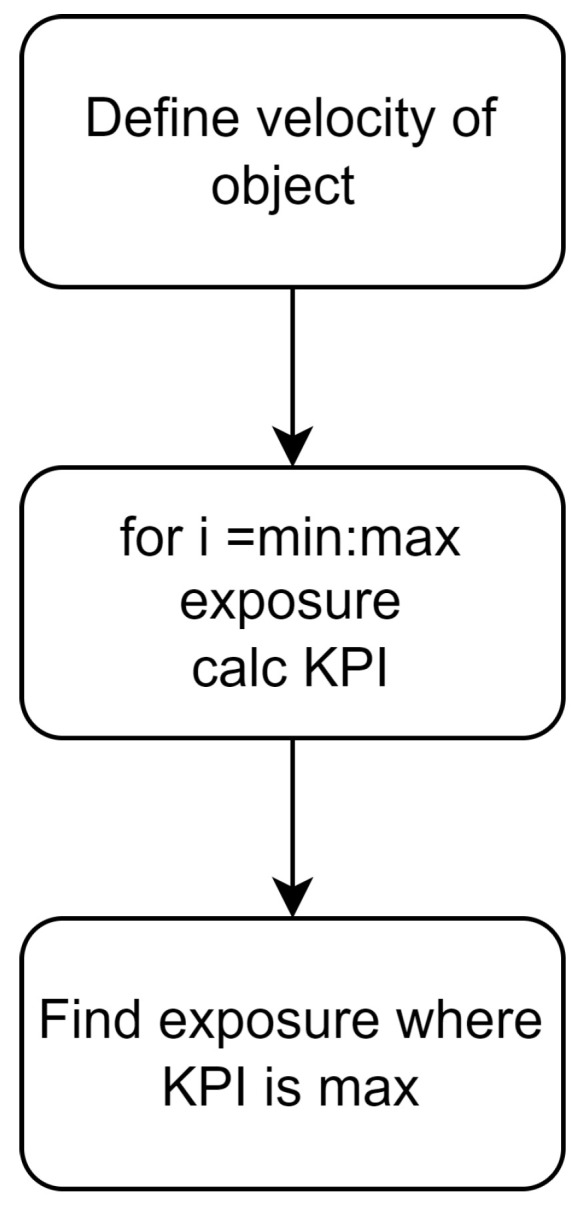
A flowchart for determining an optimal exposure time for max KPI.

Steps are then taken to optimize exposure time based on the KPIs of interest; this is outlined in more detail in Figure 8. The critical factors relating to motion blur will have to be considered. This includes the speed of objects in the scene and the frame rate required to capture the objects, the minimum frame rate will determine the maximum exposure time.

Image quality analysis is carried out on the images generated by the simulation using Imatest software [38].

**Figure 9:**
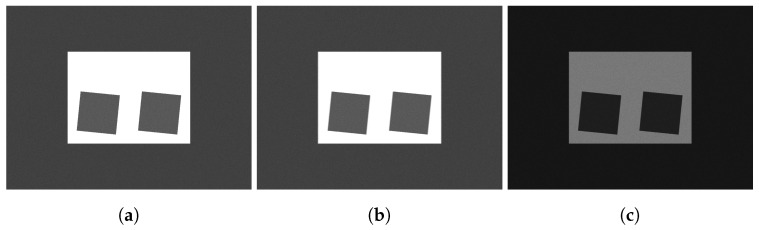
Examples of the slanted edge chart outputted from the simulation. Simulated with the following configurations: (**a**) 50 lux illumination, 0DX (static), 30 ms exposure time (**b**) 50 lux, 1000DX, 30 ms (**c**) 50 lux, 1000DX, 10 ms.

**Figure 10:**
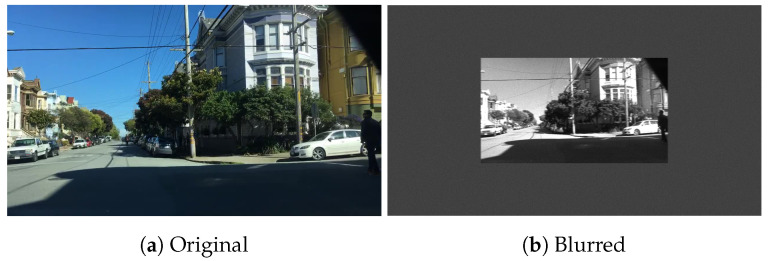
Example image from BDD dataset, blurred with horizontal movement of 350 pixels per second, which corresponds to approx. 50 kph object speed (350 pixels/second movement).

**Figure 11:**
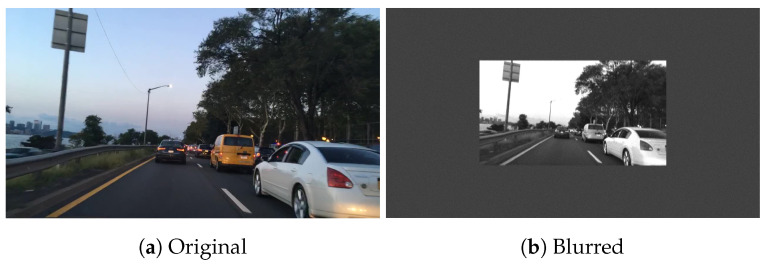
Example image from BDD dataset, blurred with vertical movement of 350 pixels per second.

Examples of the output from the simulation, where the input is the standard slanted edge chart from Imatest [37], are shown in Figure 9. Figure 10 and Figure 11 show examples of simulation output using images from the BDD dataset [64]. Figure 10 is horizontally blurred to emulate a speed of approximately 50 kmph (350 pixels/second movement). Figure 11 is vertically blurred to emulate a speed of approximately 50 kmph.

## 4. Results

This section is organized as follows. Section 4.1 presents a discussion of the effects of exposure time and motion blur on low-level image quality metrics. Section 4.2 presents an evaluation of the effects of changes in exposure time and motion blur on machine vision performance, using object detection of a stop sign as an example use case.

### 4.1. Image Quality Metric Analysis

#### 4.1.1. MTF

**Figure 12:**
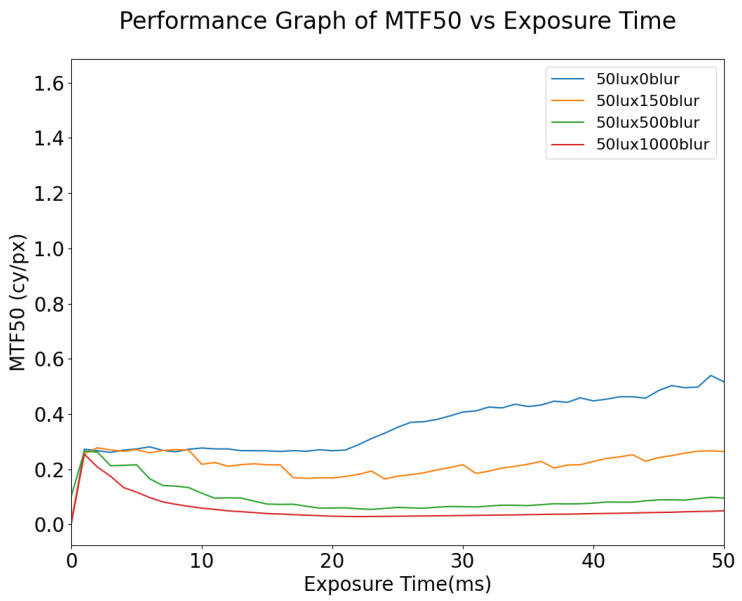
MTF50 vs. Exposure Time, simulated at 50 lux with different degrees of motion blur (DX). Note: “150blur” corresponds to 150 horizontal pixels per second (150DX).

**Figure 13:**
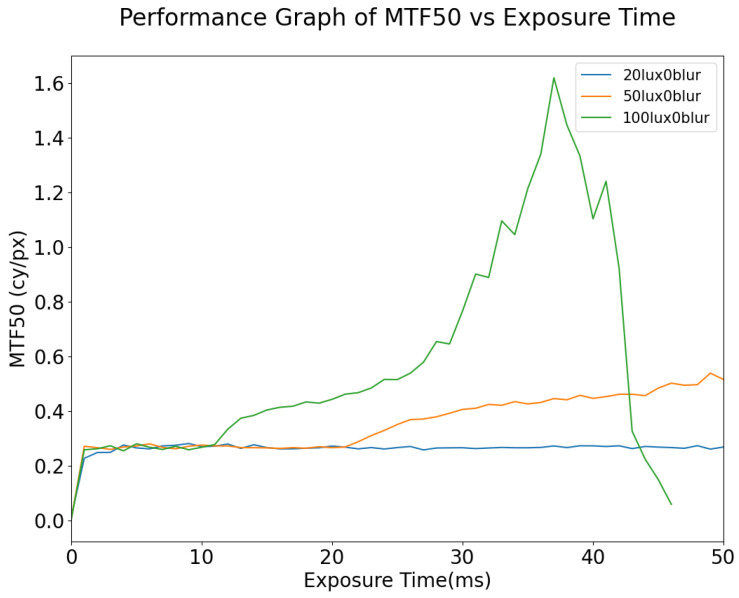
MTF50 vs. Exposure Time, for static images at different lux levels.

**Figure 14:**
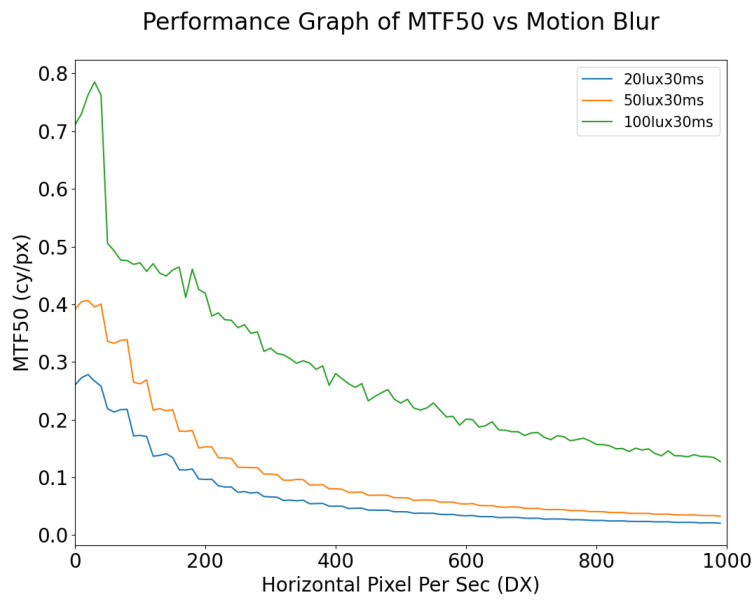
MTF50 vs. Motion Blur simulated at 30 ms exposure but at different lux levels.

Although MTF50 is one of the most common metrics used in the automotive field, changes in MTF50 can be small relative to the physical changes in the images as a result of motion blur. This suggests that MTF50 is not in itself an adequate metric for predicting computer vision performance as exposure time and/or motion blur varies. As shown in Figure 12, for static images, the MTF will increase with the exposure time; however, at an exposure time of 40 ms the image is already beginning to become overexposed while the MTF50 is still increasing, especially for lower illumination values. Another observation is that, for dynamic scenes, it is not feasible to improve the signal quality by means of increased exposure time, especially in the case where there is any significant motion blur in the scene; increasing the exposure time when the targets in the scene are moving at high speed will only increase the number of blurred pixels on the image. As shown in Figure 13, increasing the lux level results in overexposure occurring at lower exposure times. Under the given illuminator and sensor characteristics, when the illumination of the simulation is set to 50 lux, the signal remains consistent until 20 ms exposure time, at which point pixels begin to approach saturation and this causes the MTF50 to become overestimated erroneously, i.e., it is not actually the case that the image quality (as measured by MTF50 increasing) is improving. This phenomenon is also described in [25]. As shown in Figure 14, in general, for a given illumination level MTF50 decays as the level of motion blur increases. The localized spike in MTF50, for 100 lux with motion blur of 20 DX, for example, is due to blurred pixels stacking on each other while not destroying the information in the image, essentially causing an apparent increase in sharpness.

#### 4.1.2. SNR

**Figure 15:**
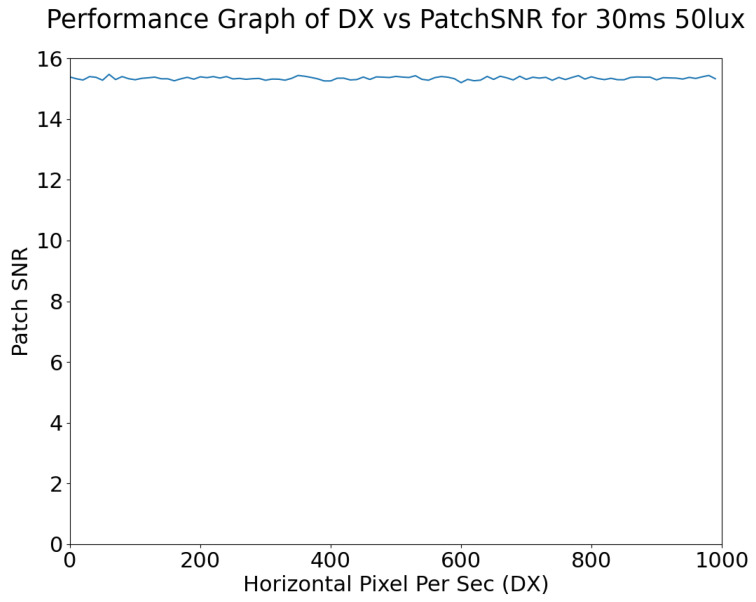
Horizontal motion blur vs. patch SNR.

Patch SNR and Edge SNR as used here are defined in Section 2.2. As shown in Figure 15, the Patch SNR is largely unaffected by motion blur; the primary cause of any variance in SNR in Figure 15 is the noise. While Patch SNR is not affected by motion blur, it is affected by exposure.

**Figure 16:**
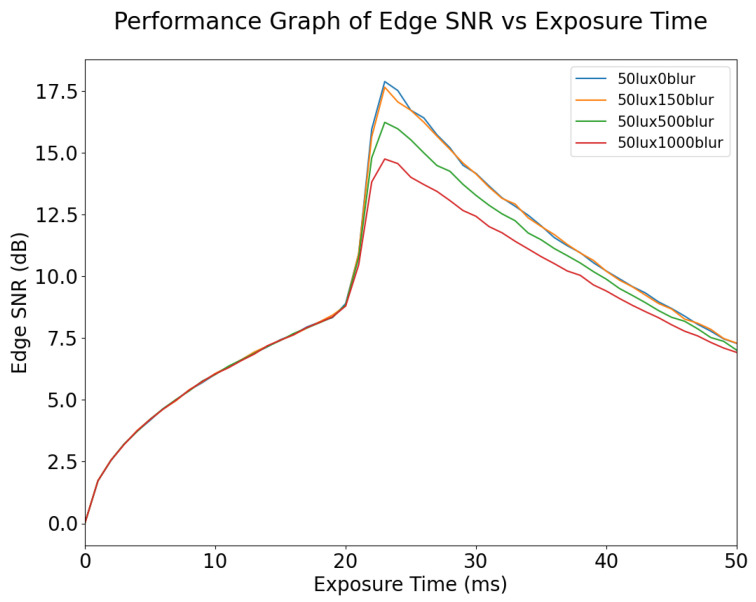
Edge SNR vs. Exposure Time for different values of blur, at 50 lux illumination. The sudden rise in Edge SNR at 20 ms exposure time is due to the pixels being saturated; when exposure time has increased to 25ms the signal component has become fully saturated.

**Figure 17:**
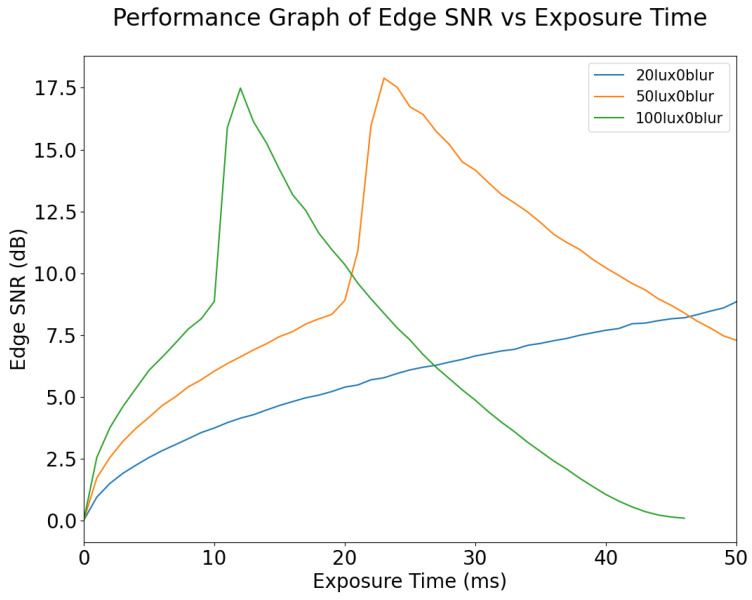
Edge SNR vs. Exposure, for different illumination levels.

The variation in Edge SNR with exposure time for different values of blur is shown in Figure 16. It can be seen that Edge SNR is affected more by exposure time than by motion blur. Edge SNR remains the same for different degrees of motion blur, while the exposure time is less than 20 ms. Edge SNR increases suddenly because of pixel saturation at 20 ms exposure time; however, this does not mean image quality has increased; rather it is caused by pixel saturation. Therefore, Edge SNR is not an ideal metric for dynamic scenes. For static scenes, Edge SNR may be a good metric for tuning exposure time up to the optimal value; however, it does not respond to motion blur for exposure time below 20 ms. As expected, increasing the ambient lighting of an image will bring the point at which overexposure and clipping occur to lower exposure times, as shown in Figure 17. This is because more photons are captured during the exposure window.

#### 4.1.3. SIC

**Figure 18:**
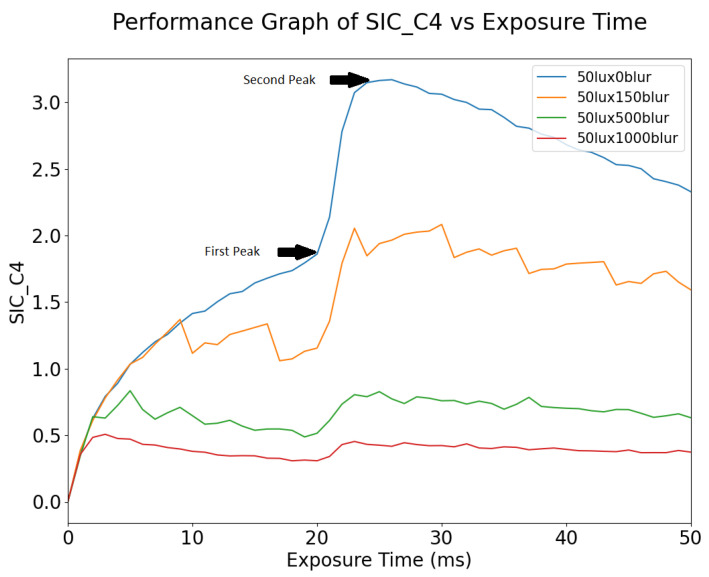
Shannon Information Capacity vs. Exposure Time for different blur values.

Figure 18 shows the variation of Shannon Information Capacity (SIC) as a function of exposure time, for different blur values. From Figure 18, the exposure time where the SIC is at its maximum is between 20 and 30 ms. After the SIC peaks, it slowly degrades as the image becomes overexposed. The signal becomes clipped, and the noise rises, thus reducing the SNR. As motion blur increases, the peak SIC value also gets smaller.

Since SIC is a measurement of potential information capacity in the image, the decrease in SIC shows that motion blur degrades the information content in the image. As motion blur increases, the benefit gained from increasing the signal level by increasing the exposure time also decreases.

As shown in Figure 18, SIC varies with both exposure time and motion blur. This suggests that it has the potential to be used as an optimization metric for dynamic scenes.

#### 4.1.4. NEQ

**Figure 19:**
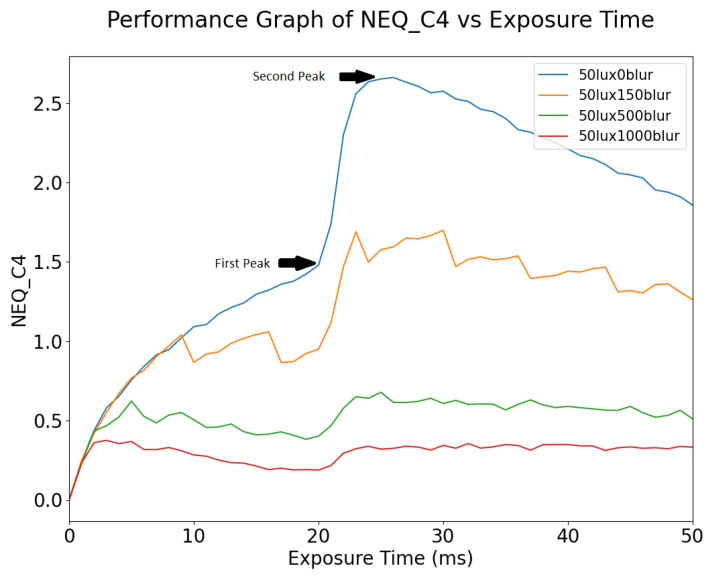
NEQ vs. Exposure Time for different blur values.

NEQ shows similar trends to SIC. From Figure 19, the exposure time where NEQ is at its maximum is between 20 and 30 ms. Like SIC, after the NEQ peaks, it slowly degrades as the image becomes overexposed. We suggest that the optimal exposure window lies between the first and second peaks of NEQ and SIC; after the second peak the signal becomes clipped and the noise continues to increase. As motion blur increases, the exposure time at which NEQ and SIC peaks drops, as does the peak value itself.

Since motion blur is a function of exposure time, as the motion blur increases, NEQ degrades earlier and more quickly. In Figure 19, the increase in motion blur affects the maximum amplitude of NEQ. This is an expected behavior, as explained above in the discussion of SIC.

Since NEQ varies for both exposure time and motion blur, it has the potential to be used as an optimization metric for dynamic images.

### 4.2. Object Detection Performance—Stop Sign

**Figure 20:**
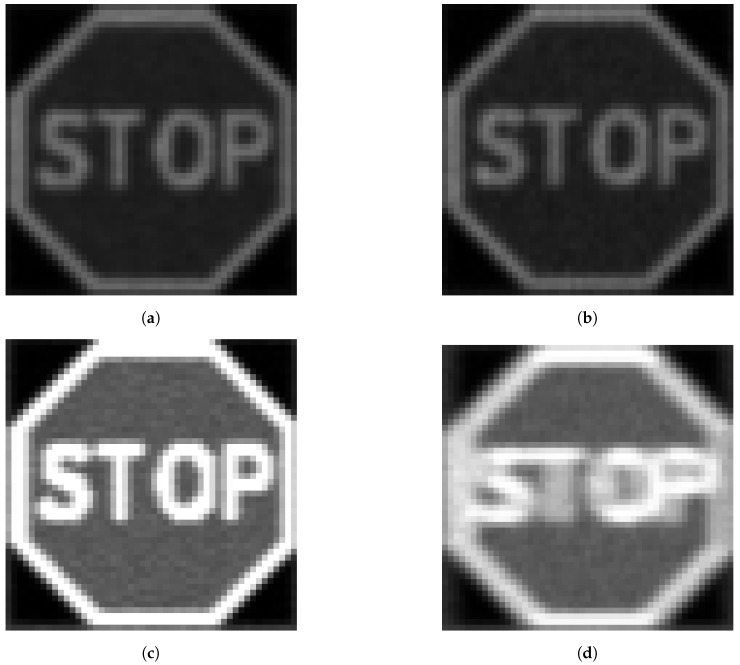
Figures (**a**,**c**) show static images of 50 × 50 px stop sign at 50 lux, and 10 ms and 30 ms exposure time, respectively. Figures (**b**,**d**) show motion blurred image 50 × 50 px stop sign at 50 lux, 200DX, and 10 ms and 30 ms exposure time, respectively. Note, that the lower exposure time in (**b**) results in less apparent blurring of the image than (**d**).

**Figure 21:**
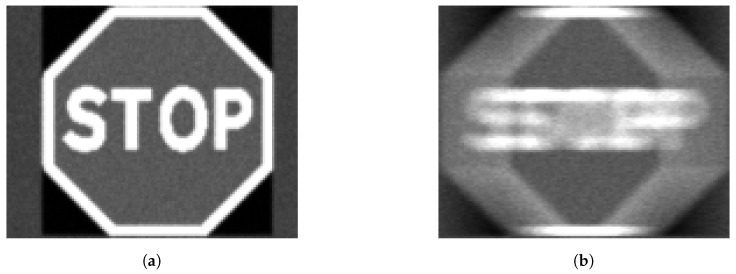
Figures (**a**,**b**) show a static and a 1000 DX version of a 100 × 100 px stop sign simulated at 50 lux and 30 ms.

An image of a stop sign is resized to 100 × 100 pixels, 50 × 50 pixels, and 32 × 32 pixels, which corresponds to large, medium, and small objects according to MS COCO [65]. The different size versions of the stop sign are inputted into the simulation tool and different degrees of motion blur and exposure time are applied. Figure 20 and Figure 21 show examples of stop signs of different sizes (small and large) at different exposure times. Visual inspection indicates that, at the same relative motion/speed, increasing the exposure will increase the degree of motion blur.

OCR and object detection algorithms are run on the output images. The results are discussed below.

#### 4.2.1. Frequency Content

A simple measure of the frequency content for large, medium, and small targets was obtained in Matlab, using the *improfile* function to obtain the pixel-value cross-sections through the center of the stop sign, to determine if it provided useful information to predict machine vision performance. A simple derivative function, shown in Equation (15), was used to find the transitions between the foreground and background:

(15) (0.5×(IMAGE[x−1]+IMAGE[x+1]))

where *x* pixel location along the 1D profile and IMAGE[x−1] is the pixel value at profile location x−1. The peaks in the derivative were found, and the distance between the peaks was measured. The distance between the peaks is referred to here as the “spatial frequency range”, i.e., where the energy in the SFR is concentrated. The inverse of this distance is the spatial frequency in cycles/pixel (cy/px).

Based on analysis of the spatial frequency range of the targets, the following points emerge:

1. For large targets, the spatial frequency range, the range of SFR values for the derivative peaks, is 0.04–0.1 cy/px. This corresponds approximately to Nyquist/4.
2. For the medium targets, the range of spatial frequencies is 0.08–0.2 cy/px. This corresponds to the MTF range between Nyquist/2 and Nyquist/4.
3. For the small target, the range of spatial frequencies is 0.125–0.3 cy/px. This corresponds approximately to Nyquist/2.

#### 4.2.2. Object Detection Performance

Object detection algorithms are run on the large, medium, and small stop signs. The algorithm used is YOLOV5m [52]. The primary reason for this choice is that YOLOv5 is a well-characterized and understood algorithm. The main objective is to optimize exposure time and investigate its impact on image quality. Consequently, employing YOLOv5m allows us to efficiently perform object detection without diverting attention and resources from our primary research goals.

**Figure 22:**
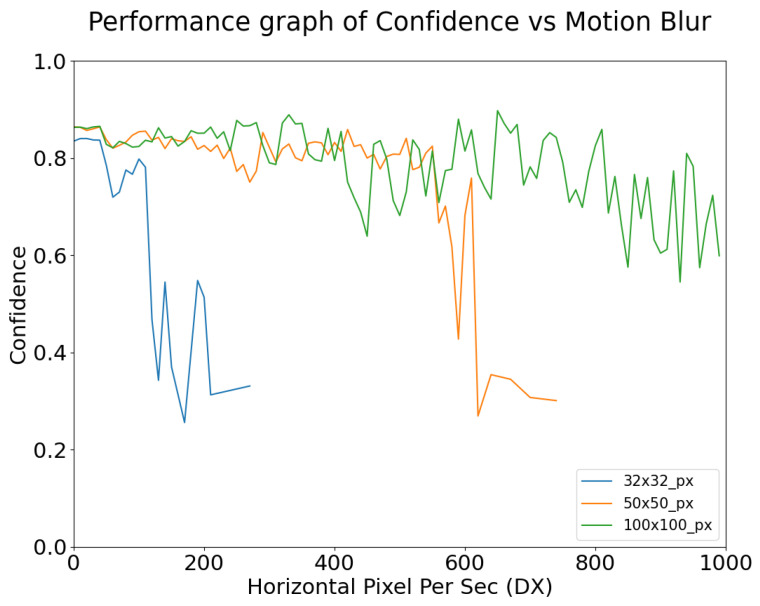
Object Detection Performance Confidence vs. Motion Blur, simulated at 30 ms exposure time and 50 lux ambient illumination, for targets of three different sizes (see legend).

**Figure 23:**
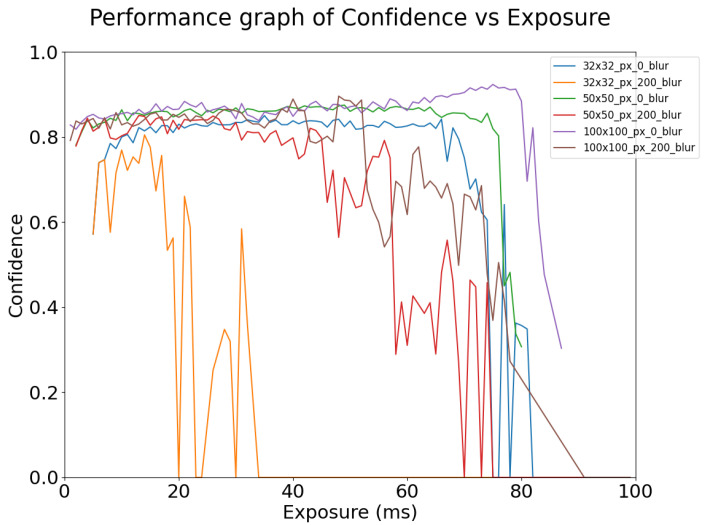
Object Detection Performance Confidence vs. Exposure Time—Simulated at 50 lux ambient illumination, for different target sizes (see legend). Results for both static (0 blur) and motion blurred (200 blur) stop signs are shown.

Figure 22 and Figure 23 show the confidence level of object detection as a function of blur, and exposure time, respectively. Each plot is shown only for the range of x-axis values where detection actually occurs (for example, for small targets in Figure 22, detection fails for relatively small values of blur). As expected, the confidence of YOLOv5m’s detection of stop signs degrades as the degree of motion blur increases.

As shown in Figure 22, object detection performance remains robust to motion blur up until a point, where the confidence begins to drastically decrease and eventually detection fails. This failure point varies depending on the size of the target and the degree of motion blur. For example, the 32 × 32 targets were detected with high confidence until approximately 200DX whereas for 50 × 50 targets the high confidence continued until approximately 600DX. This point may also be affected by the use of different algorithms.

As shown in Figure 23, for object detection algorithms to function, the image must be adequately exposed according to the target size, and the signal must be above the noise floor. For example, the 100 × 100 pixel targets can be detected with confidence at an exposure time of 1 ms whereas the 32 × 32 pixel targets are not detected until the exposure time is 5 ms. This also shows that smaller targets require more contrast than larger targets to be detected. However, as the exposure time increases to the point where the image is saturated and clipping occurs, the object detection confidence begins to decrease and eventually, detection fails; this is particularly apparent in the right-hand side of the figure.

From Figure 23, for the 32 × 32 pixel targets, when a 200 pixel per second blur is applied, the exposure window within which high performance is obtained ranges from 75 ms down to 20 ms. For the 50 × 50 pixel targets, when applied with the same amount of blur, the exposure window range shrinks from 80 ms to 70 ms. For the 100 × 100 pixel targets, when applied with the same amount of blur, the exposure window range shrinks from 84 to 78 ms. This behavior is due to a single-pixel change in a small target having a larger proportional effect than a single-pixel change in a large target. Hence, a 200 pixels per second blur will degrade a larger percentage of information in a small target than a large target. In the case of the small target, there are more than 60 pixels displaced at 30 ms exposure, which means the displacement is greater than the target size.

**Figure 24:**
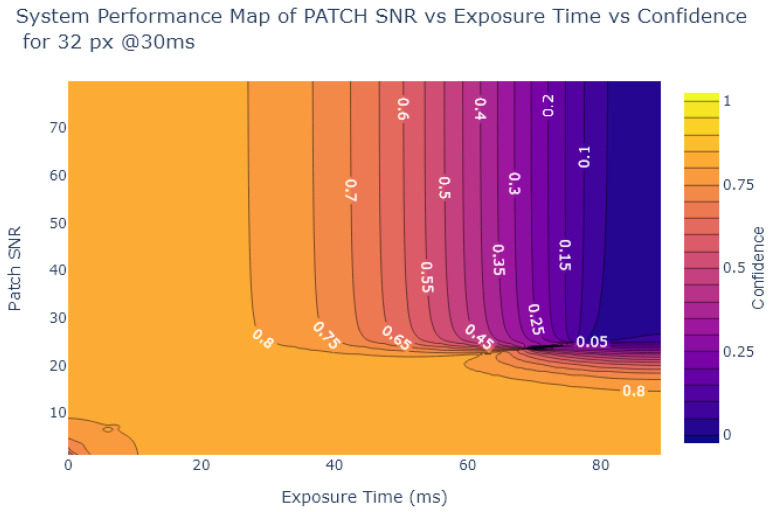
System Performance Map of Patch SNR vs. Exposure vs. Object Detection Confidence for 32 × 32 stop signs.

**Figure 25:**
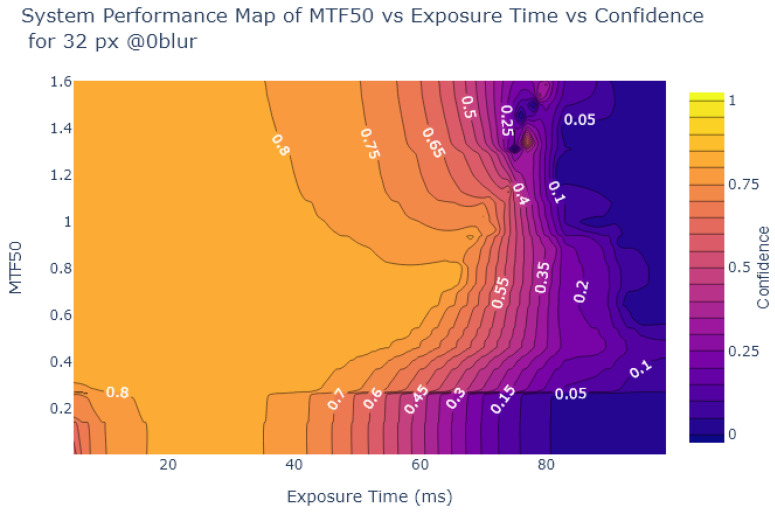
System Performance Map of MTF50 vs. Exposure vs. Object Detection Confidence for 32 × 32 stop signs.

**Figure 26:**
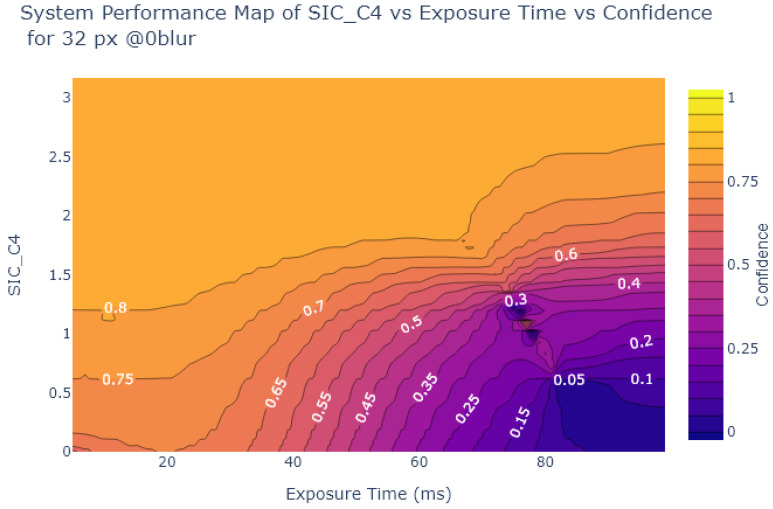
System Performance Map of SIC vs. Exposure vs. Object Detection Confidence for 32 × 32 stop signs.

**Figure 27:**
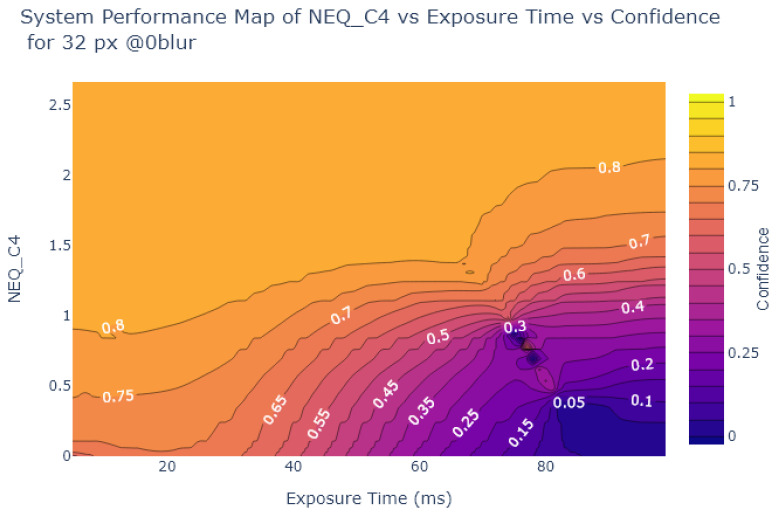
System Performance Map of NEQ vs. Exposure Time vs. Object Detection Confidence for 32 × 32 stop signs.

**Figure 28:**
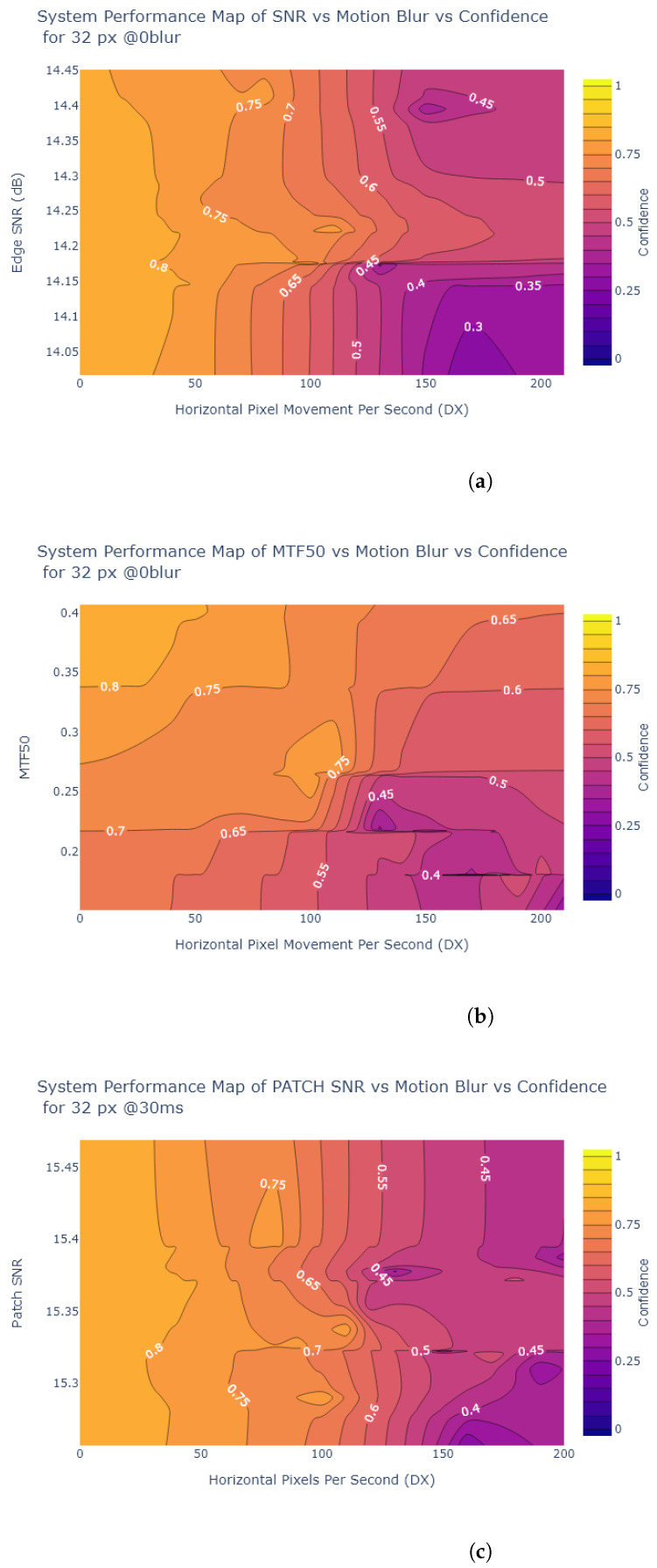
SPM of (**a**) Edge SNR (**b**) MTF50 (**c**) Patch SNR vs. Motion Blur vs. Object Detection Confidence for 32 × 32 pixel STOP signs at 30 ms. These graphs show that the correlation between the metrics and confidence is poor, most of the correlation lies between motion blur and confidence.

Inspired by Liu et al. [54], the object detection confidence is also plotted in a contour plot against exposure and various image quality metrics, to create System Performance Maps (SPM). The values of the metrics at each exposure time are obtained from the analysis in Section 2.2. Figure 24, Figure 25, Figure 26 and Figure 27 show SPMs as a function of exposure time, while Figure 28 shows SPMs as a function of blur. Small (32 × 32 px) and large targets (100 × 100 px) are considered the extremes of the size range. In the interests of brevity, the plots shown are for 32 × 32 pixel targets only because there is a significant change in object detection performance for these small targets, whereas detection of the 100 × 100 pixel targets is quite robust, as can be observed in Figure 22 and Figure 23.

Figure 24 and Figure 25 indicate that traditional metrics like Patch SNR and MTF50 are poor predictors of object detection performance as a function of exposure time. This is indicated by the presence of near vertical lines in the SPMs of these metrics, showing that there are points where the metric value does not correlate well with object detection confidence, particularly evident in Figure 24.

On the other hand, as shown in Figure 26 and Figure 27, the alternative metrics like SIC and NEQ show a stronger relationship with object detection performance. For example, as shown in Figure 26, when the SIC is low and the image is overexposed, then the detection confidence is also low. From Figure 26, the range of exposure values where SIC provides useful information can be deduced, using the commonly-used object detection standard of a confidence level of at least 0.6 as a useful performance threshold [66]. For object detection confidence to be above 0.6, if exposure is above 5 ms and below 30 ms, then the SIC value is not as relevant. But if the exposure exceeds 30 ms we can use the SPM to deduce the required SIC to maintain object detection performance, in this case, an SIC of over 1.5. Similarly, in Figure 27, a usable range of exposures for NEQ can be determined. Using the same confidence threshold of 0.6, a similar exposure range of 5ms to 30ms is obtained.

**Figure 29:**
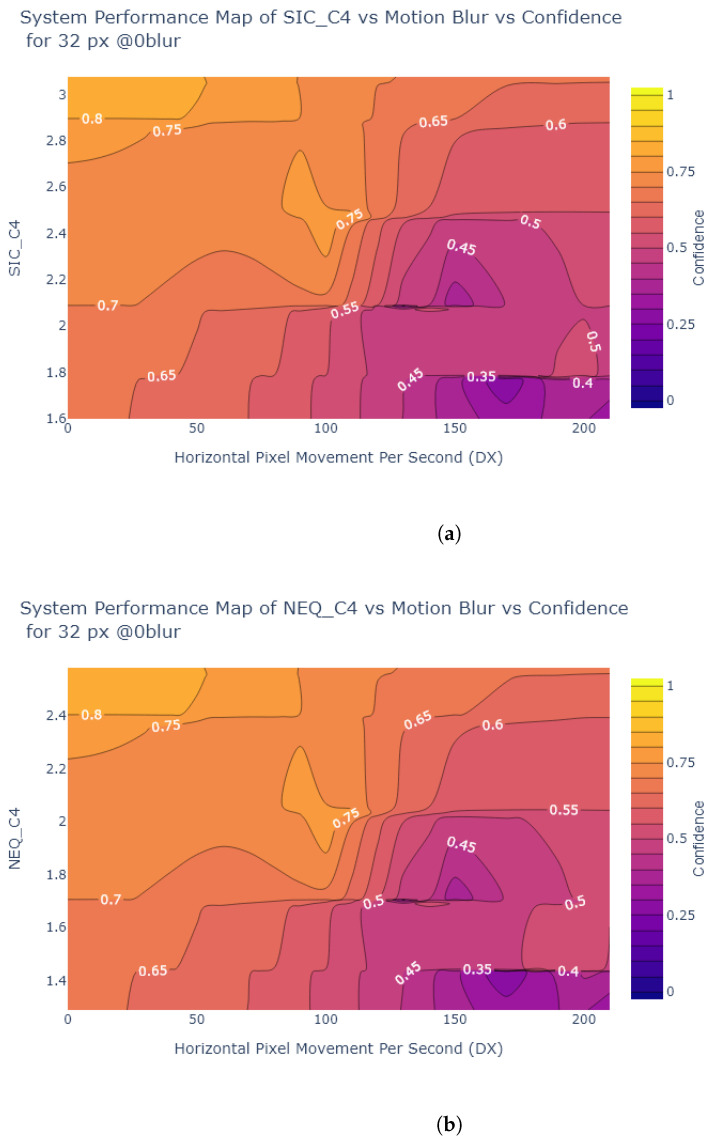
SPM of (**a**) SIC (**b**) NEQ vs. Motion Blur vs. Object Detection Confidence for 32 × 32 pixel STOP signs at 30 ms. Once again the graph suggests an inversely proportional relationship between motion blur and confidence, i.e., as motion blur increases, confidence decreases.

As shown in Figure 28 and Figure 29, all of the metrics indicated have poor correlation with object detection performance as motion blur varies. For example, when motion blur increases to above 100 DX, in the case of 30 ms exposure time this corresponds to 3 px displacement, approximately 10 % displacement of the 32 × 32 target. At this point, an increase in the metric value does not correspond to an increase in object detection confidence.

Examining the frequency content for stop signs of different sizes (based on the simple derivative method described in Section 4.2.1), a strong correlation is not observed between computer vision performance and MTF at the frequencies corresponding to Nyquist, Nyquist/2, and Nyquist/4.

#### 4.2.3. Optical Character Recognition (OCR)

As an additional test, an OCR algorithm is used to detect the letters on large, medium, and small stop signs. The algorithm used is Paddle OCR, also called PP-OCRv3 [67].

**Figure 30:**
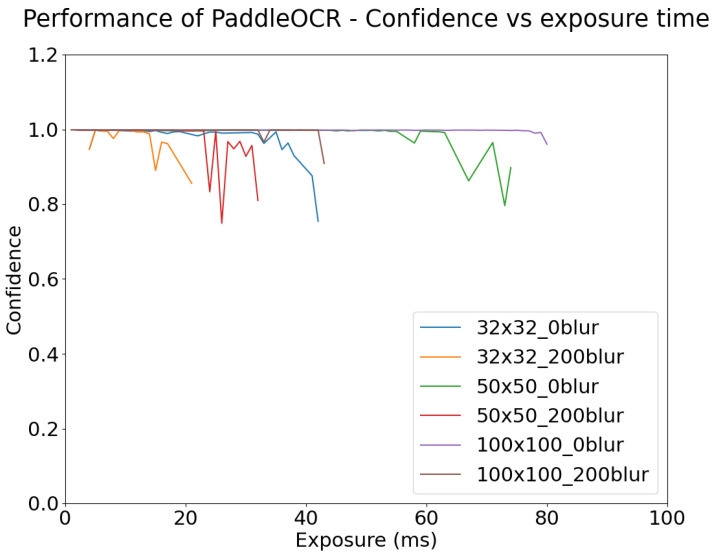
Paddle OCR Confidence vs. Exposure Time for different target sizes, for different blur values (see legend).

**Figure 31:**
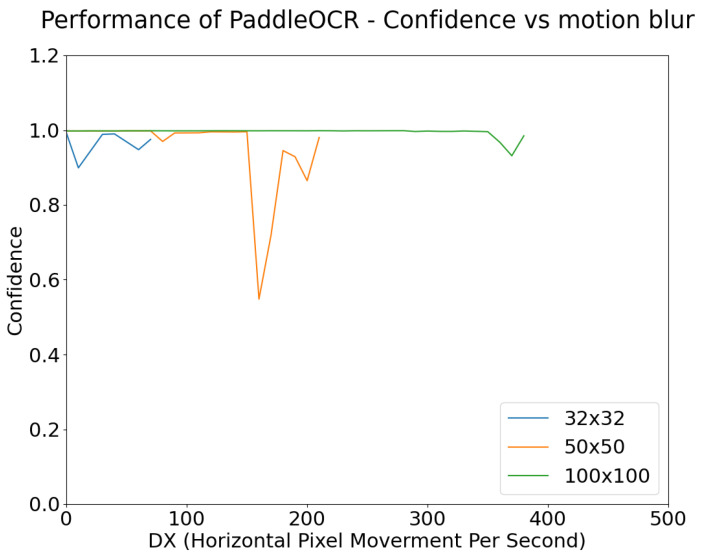
Paddle OCR Confidence vs. Motion Blur for different target sizes, with fixed 30 ms exposure.

Figure 30 and Figure 31 show the change in OCR confidence versus motion blur and exposure. As for Figure 22 and Figure 23, the graphs in Figure 30 and Figure 31 are only plotted until the word “STOP” is no longer recognized correctly. After the detection of STOP fails, it was observed that the algorithm begins to misclassify STOP as other text, but its confidence is very low. This indicates that information that contributes to OCR is degraded by overexposure and blur.

As shown in Figure 30, OCR starts to work when the exposure time reaches a certain minimum, which varies depending on the circumstances, for example, for a small target with no blur, this is 4 ms, while for a medium target with a blur of 200 pixels/second, it is 2 ms. However, as exposure time increases, as seen previously it reaches a point where the image is saturated and clipping occurs, OCR confidence begins to decrease, and eventually, OCR fails. Larger targets are easier to detect, for example, 100 × 100 pixel targets can be detected with confidence at an exposure of 1 ms, whereas the 32 × 32 pixel targets are not detected until the exposure time is 4 ms; smaller targets require more contrast than larger targets for detection.

As also shown in Figure 30, smaller targets are more affected by motion blur than larger targets. For the 32 × 32 pixel targets, when a blur of 200 DX is applied, the performance window falls from 40 ms to 22 ms. For 50 × 50 pixel targets, when applied with the same amount of blur, the exposure window ranges from 75 to 32 ms. For the 100 × 100 pixel targets, when applied with the same amount of blur, the exposure window ranges from 80 ms to 43 ms. As before, this behavior is due to changes in a small target having a larger proportional change than in a large target. This is further seen in Figure 31, where for a standard image of 30 ms exposure, 100DX is enough motion blur for the OCR of small targets to fail, while for large targets, the OCR performance remains high even for blur values as high as 350DX.

## 5. Discussion

The study reveals that while changes in exposure time and motion blur significantly affect image quality metrics, the correlation with machine vision performance is not straightforward. The experiments on image quality metrics described in Section 4.1 indicate that metrics such as SIC and NEQ may be more useful than MTF and SNR in terms of their ability to capture changes due to motion blur and exposure time. As observed from the experiments, care is needed in interpreting the results, for example, the choice of optimal exposure time depends on selecting appropriate points in the graph of NEQ vs. exposure time in Figure 19. The effects of image over-exposure (with increasing exposure time) have also been found to be quite significant. From the experiments described in Section 4.2, object detection algorithms and OCR algorithms both exhibit robustness to variation in exposure time, maintaining a large window of high performance for large static objects. Target size has an influence on performance. The use of System Performance Maps allows a more detailed analysis of the correlation between metrics and object detection performance. As might be expected, smaller objects are impacted much more significantly. In general, motion blur is a more significant challenge than exposure time; this is especially apparent in smaller targets which are more sensitive to pixel displacement. Traditional image quality metrics such as MTF do not correlate well with computer vision performance. Newer metrics like SIC and NEQ showed better, albeit non-linear, correlations, highlighting the need for a combination of metrics to accurately predict performance. The study underscores the importance of exploring multiple metrics to optimize camera systems effectively. By understanding the behavior and different sensitivities of different metrics, we can better address the complex requirements of autonomous vehicle applications.

## 6. Conclusions and Future Works

This paper has presented a comprehensive methodology to optimize camera exposure time for automotive applications, providing a simulation tool for faster and more efficient prototyping of camera systems. The simulation tool is the first of its type to incorporate a physics-based noise and blur model. This approach will allow the user to rapidly find the optimal exposure window for a given scenario.

The results demonstrated that while object detection algorithms perform well across a range of exposure times, motion blur significantly degrades performance, especially for smaller targets. Traditional metrics like MTF proved inadequate in terms of predicting computer vision performance, whereas newer metrics like SIC and NEQ showed stronger correlations. The study also examined the impact of target size on the correlation between image quality metric and computer vision performance.

Overall, this study suggests that the answer may not be a single metric, but rather a combination of multiple metrics, with different contributions.

Future work may include extending the simulation to cater to rolling shutter cameras and localized motion blur. With the help of semantic segmentation algorithms, object-level localized simulation may be achieved. This will allow for the simulation and blurring of individual objects according to the proposed speed of the target to allow more detailed insights to be derived from the simulation. A more complex blur kernel will allow the simulation to accommodate various other types of blur that may occur in an image, for example, simulating situations such as a camera mounted on a car going over a speed bump. Incorporating a more intricate lens model would enable the simulation of out-of-focus blurring and provide a more precise representation of the various lens types mountable on a camera. In the next version of the simulation, we will incorporate the functionality for multi-exposure HDR imaging, as well as accommodate other more complex color spaces. We also plan a more detailed analysis of the deep learning component, where the effects of motion blur and exposure time on different architectures will be investigated. We will also explore the possibility of improving object detection performance by fine-tuning the algorithms with motion-blurred images and evaluating the changes in performance. There is potential to introduce image quality metrics from other fields of research, such as the medical imaging domain, for example, Contrast-to-Noise Ratio (CNR) [30,68] and Ideal Observer SNR (SNRI) [69].

## Figures and Tables

**Table 1:** Image quality metrics considered in this study, and their units.

Image Quality Metrics	Units
SNR	dB
MTF50	Cycles per pixel
SIC	Bits per pixel
NEQ	Photons

**Table 2:** Calculation of Motion Blur. This table is generated on the assumption of the following: given a 4096 × 2160 camera resolution with a 99-degree horizontal and 63-degree vertical field of view. This yields a 41-pixel per degree, pixel representation in the image. The typical size of a car is assumed to be 4.9 × 1.8 m. The typical size of a human is assumed to be 0.5 × 1.75 m.

Distance	Target Speed	Target Speed	Target Width	Target Height	Exposure Time	Estimated Motion Blur
(m)	(kmph)	(${\mathrm{ms}}^{-1}$)	(m)	(m)	(ms)	(Pixels)
25	10	2.778	0.5	1.75	15	3.938
50	10	2.778	0.5	1.75	15	1.969
100	10	2.778	0.5	1.75	15	0.984
100	20	5.556	0.5	1.75	15	1.969
100	20	5.556	4.9	1.8	30	3.94
100	50	13.889	4.9	1.8	30	9.84
50	100	13.889	4.9	1.8	15	19.675
50	100	13.889	4.9	1.8	30	39.35
100	100	27.778	4.9	1.8	30	19.69

## Data Availability

The original contributions presented in the study are included in the article.
